# Liver fat accumulation is associated with reduced hepatic insulin extraction and beta cell dysfunction in healthy older individuals

**DOI:** 10.1186/1758-5996-6-43

**Published:** 2014-03-26

**Authors:** Francis M Finucane, Stephen J Sharp, Mensud Hatunic, Alison Sleigh, Ema De Lucia Rolfe, Avan Aihie Sayer, Cyrus Cooper, Simon J Griffin, Nicholas J Wareham

**Affiliations:** 1 MRC Epidemiology Unit, Institute of Metabolic Science, University of Cambridge, Addenbrooke’s Hospital, Box 285, Hills Road, Cambridge CB20QQ, UK; 2 Galway Diabetes Research Centre, HRB Clinical Research Facility, School of Medicine, NUI Galway, Galway, Ireland; 3 Institute of Metabolic Science, University of Cambridge Metabolic Research Laboratories, Cambridge CB20QQ, UK; 4 Wolfson Brain Imaging Centre, University of Cambridge, Cambridge CB20QQ, UK; 5 MRC Lifecourse Epidemiology Unit, University of Southampton, Southampton SO166YD, UK

**Keywords:** Adaptation index, Beta cell dysfunction, C-peptide-genic index, Disposition index, Hepatic insulin extraction, Insulinogenic index, Intrahepatic lipid, Non-alcoholic fatty liver disease

## Abstract

**Background:**

There is a well-established association between type 2 diabetes and non-alcoholic fatty liver disease (NAFLD) secondary to excess accumulation of intrahepatic lipid (IHL), but the mechanistic basis for this association is unclear. Emerging evidence suggests that in addition to being associated with insulin resistance, NAFLD may be associated with relative beta-cell dysfunction. We sought to determine the influence of liver fat on hepatic insulin extraction and indices of beta-cell function in a cohort of apparently healthy older white adults.

**Methods:**

We performed a cross-sectional analysis of 70 healthy participants in the Hertfordshire Physical Activity Trial (39 males, age 71.3 ± 2.4 years) who underwent oral glucose tolerance testing with glucose, insulin and C-Peptide levels measured every 30 minutes over two hours. The areas under the concentration curve for glucose, insulin and C-Peptide were used to quantify hepatic insulin extraction (HIE), the insulinogenic index (IGI), the C-Peptide increment (CGI), the Disposition Index (DI) and Adaptation Index (AI). Visceral fat was quantified with magnetic resonance (MR) imaging and IHL with MR spectroscopy. Insulin sensitivity was measured with the Oral Glucose Insulin Sensitivity (OGIS) model.

**Results:**

29 of 70 participants (41%) exceeded our arbitrary threshold for NAFLD, i.e. IHL >5.5%. Compared to those with normal IHL, those with NAFLD had higher weight, BMI, waist and MR visceral fat, with lower insulin sensitivity and hepatic insulin extraction. Alcohol consumption, age, HbA1c and alanine aminotransferase (ALT) levels were similar in both groups. Insulin and C-Peptide excursions after oral glucose loading were higher in the NAFLD group, but the CGI and AI were significantly lower, indicating a relative defect in beta-cell function that is only apparent when C-Peptide is measured and when dynamic changes in glucose levels and also insulin sensitivity are taken into account. There was no difference in IGI or DI between the groups.

**Conclusions:**

Although increased IHL was associated with greater insulin secretion, modelled parameters suggested relative beta-cell dysfunction with NAFLD in apparently healthy older adults, which may be obscured by reduced hepatic insulin extraction. Further studies quantifying pancreatic fat content directly and its influence on beta cell function are warranted.

**Trial registration:**

ISRCTN60986572

## Background

Excess accumulation of intrahepatic lipid (IHL) leads to non-alcoholic fatty liver disease (NAFLD), an important component of the spectrum of metabolic abnormalities implicated in the pathogenesis of type 2 diabetes mellitus [1]. The liver is the primary site of insulin clearance in humans [2]. Previous studies have suggested that liver fat accumulation is associated with absolute increases in insulin secretion from the beta-cell, in order to compensate for insulin resistance and maintain euglycaemia [3].

Beta-cell dysfunction per se is not generally considered a complication of NAFLD [1,4]. However, several studies suggest that elevated liver fat is associated with reduced hepatic insulin extraction (HIE) [5,6]. Thus, it is plausible that IHL accumulation could be associated with a relative beta-cell failure to adapt to increasing insulin resistance but that such a defect might not be apparent, because the concomitant reduction in HIE would lead to elevated circulating insulin levels. Indeed, the accumulation of ectopic fat in the pancreas is increasingly recognised as a cause of beta-cell dysfunction [7]. While a study of 64 overweight white adults with a family history of type 2 diabetes found an association between pancreatic fat content and glucose tolerance status, no such association was found specifically for measures of beta cell function [8]. More recently, in a study of almost 1000 Chinese young adults (mean age 21 years), alanine aminotransferase (ALT, a marker of hepatic steatosis) was associated with beta-cell dysfunction. However, a direct association with liver fat content (measured with computerised tomography) and beta-cell dysfunction was not found [9].

We sought to determine whether IHL accumulation was associated with altered indices of beta-cell function, including those which take account of glucose excursion and insulin sensitivity in a cohort of healthy older people. A second objective was to determine the influence of HIE on the relationship between IHL and those indices, by comparing insulin-derived beta-cell function measures with those derived from C-Peptide measures. For this, we conducted a post-hoc, cross- sectional analysis of metabolic and anthropometric data from a cohort of healthy older adults who participated in the Hertfordshire Physical Activity Trial (HPAT) [10].

## Methods

The rationale and design for the Hertfordshire Physical Activity Trial (ISRCTN 60986572) have been described previously [10]. Data reported here relate to post-hoc, cross-sectional analyses of volunteers’ anthropometric and metabolic characteristics at the time of their entry into the study. Each participant provided written informed consent. The original study protocol was approved by the Hertfordshire Research Ethics Committee (LREC ref. 05/Q0201/23).

Trial participants were recruited from the Hertfordshire Cohort Study, consisting of men and women born in Hertfordshire, UK between 1931-39 and still residing there [11]. Specifically, those who were deemed to be potentially suitable by their general practitioner for inclusion in a supervised aerobic exercise programme and who lived within ten miles of the exercise facility were invited to participate, as described previously [10]. Those with known diabetes, untreated or unstable ischaemic heart disease or any medical condition that would preclude participation in an exercise programme were excluded from the trial. However, participants with incident diabetes (diagnosed at the time of entry to this study) were included in these analyses. Recruits attended the clinical research facility after an overnight fast. Of 106 who attended the screening visit, six were deemed to be unsuitable for the study because of poor mobility, pre-existing diabetes, symptoms or signs suggestive of untreated ischaemic heart disease or a combination of these factors and were excluded. Of the remaining 100, MR imaging and spectroscopy was not performed in 30 individuals who had claustrophobia, cardiac pacemakers or metal implants. Thus, 70 participants who enrolled in the study had baseline liver spectroscopy measures performed and constitute the cohort described herein.

All measurements were undertaken by trained staff adhering to standard operating procedures. Weight was measured on a Tanita® (Tokyo, Japan) scale and height with a Seca® (Hamburg, Germany) wall-mounted stadiometer. Waist circumference was measured using a D-loop non-stretch fibreglass tape measure and defined as the midpoint between the lower costal margin and the level of the superior iliac crests. Magnetic resonance measures of intrahepatic lipid (IHL) and visceral adipose tissue (VAT) were conducted on a whole body Siemens 3T Tim® Trio scanner (Erlangen, Germany), as described previously [10]. A questionnaire was used to quantify alcohol consumption in units per week.

A standard 75 g oral glucose tolerance test (OGTT) was performed. Fasting samples were taken for glucose, insulin and C-peptide levels. Glucose was measured using a hexokinase assay (Siemens, Frimley, UK). Insulin and C-Peptide were measured using a fluorometric autoDELFIA immunoassay (Perkin Elmer Life Sciences, Turku, Finland). After ingestion of glucose, further samples were taken every 30 minutes over two hours. Samples for glucose and lipid profiles were processed immediately while those for insulin and C-peptide were spun and frozen for subsequent batch analysis. All samples were processed in the same laboratory. The Oral Glucose Insulin Sensitivity (OGIS) model [12] was used to determine peripheral insulin sensitivity based on dynamic insulin and glucose responses during the OGTT.

We derived a composite metabolic risk score (zMS, incorporating the individual components of the metabolic syndrome) by standardising and then summing the following variables: (systolic blood pressure + diastolic blood pressure)/2, log 2-hour plasma glucose, log fasting insulin, inverted fasting HDL-cholesterol, log triglyceride and waist circumference, as previously described [13,14]. The component variables were standardised using sex-specific means and standard deviations from the larger Hertfordshire Cohort Study population (n = 3000), from which these participants were recruited, excluding those with prevalent diagnosed diabetes.

The areas under the concentration curve (AUC) for glucose, insulin and C-Peptide during the OGTT were calculated with the trapezium rule. Hepatic insulin extraction (HIE), a measure of the proportion of insulin secreted from the pancreas which is removed by the liver prior to entering the systemic circulation, was derived from the difference in the AUCs for insulin and C-Peptide using the equation [1 - (AUCinsulin/AUCC - Peptide)]. The insulinogenic index (IGI), quantifying the suprabasal insulin increment relative to the corresponding glucose increment within 30 minutes of oral glucose loading was calculated from [*Δ*Insulin 30/*Δ*Glucose 30] [15,16]. In other words, the IGI provides an estimate of changes in insulin levels in the context of how glucose levels are also changing within 30 minutes of glucose ingestion. The equivalent index for quantifying the relative C-Peptide increment (CGI) was calculated using [*Δ*C - Peptide 30/*Δ*Glucose 30]. In order to assess the ability of beta cells to adapt insulin secretion to changes in insulin sensitivity, we derived two additional indices, the Disposition Index (DI, calculated as the product of IGI and OGIS) and the Adaptation Index (AI, the product of CGI and OGIS) [17]. So, in addition to taking changes in glucose levels into account when considering the relevance of insulin and C-Peptide responses after glucose ingestion, these latter two indices further take account of whole body insulin sensitivity.

To study the association between IHL as an exposure and these various measures of beta-cell function as outcomes, linear regression was used including IHL as a continuous exposure and adjusted for age, sex, MRI-visceral fat area and alcohol consumption. In order to help to illustrate the differences between those with normal versus high liver fat content and purely for presentational purposes, means and standard errors for each outcome have been described in tables and figures separately for participants with IHL ≤ 5.5% and with IHL >5.5%, the latter being the generally accepted, albeit arbitrarily defined threshold for diagnosing NAFLD [18]. However, in all of our statistical analyses, IHL was treated as a continuous rather than a categorical variable.

## Results

Of the 70 HPAT participants included in these analyses, 39 were men. Three participants (4.3%) were found to have incident, asymptomatic type 2 diabetes based on OGTT results and were included in all analyses. A further 27 (38.6%) had non-diabetic hyperglycaemia, i.e. either impaired fasting glucose, impaired glucose tolerance or both. The median IHL content was 3.6% (range 0.2- 34.5%) while 42% of participants had IHL >5.5%, thus exceeding our arbitrary threshold for NAFLD [18]. Data relating to anthropometric and metabolic characteristics of the cohort and how these differed between those with normal versus elevated IHL are summarised in Table 1. As anticipated, those with NAFLD had increased indices of adiposity and decreased insulin sensitivity and hepatic insulin extraction compared to those with normal liver fat content, with a higher ALT, composite metabolic risk score and worse lipid profiles. There was no difference in age, alcohol consumption, smoking status, diabetes family history, medication usage, blood pressure, total cholesterol or HbA1c between the groups.

**Table 1:** Characteristics of study participants with normal liver fat content (IHL ≤ 5.5%) versus those with elevated liver fat (IHL > 5.5%)

**Variable**	**IHL ≤5.5%**		**IHL >5.5%**		**p**
	**Mean**	**± SD**	**Mean**	**± SD**	**p**
M:F	20:21	-	19:10	-	
Age (years)	71.5	2.6	71.0	2.2	0.48
Weight (Kg)	70.1	11.3	84.4	12.7	<0.001
BMI (kg m^-2^)	25.2	2.7	28.6	3.6	<0.001
Waist (cm)	91.1	10.2	104.6	9.4	<0.001
MRI VAT (cm^2^)	93.1	48.5	176.5	55.9	<0.001
Alcohol (units/week)*	2.0	(1.0,6.0)	2.0	(0,14.0)	0.88
ALT (iu/l)*	21.0	(17.0,27.0)	32.0	(24.0,36.0)	<0.001
HbA1c (%)	5.6	0.3	5.7	0.3	0.20
HIE (%)	88.1	2.8	85.6	4.5	0.01
OGIS (ml min^-1^ m^-2^)	438.6	52.1	367.8	52.4	<0.001
IHL (%)*	1.8	(0.9,3.4)	14.6	(7.9,19.0)	<0.001
SBP (mmHg)	135.4	18.0	139.2	17.0	0.38
DBP (mmHg)	74.2	8.8	76.0	10.2	0.44
Fasting Glucose (mmol/l)	4.9	0.4	5.2	0.5	0.028
2-hour Glucose (mmol/l)	6.5	1.7	8.2	1.8	<0.001
Fasting Insulin (pmol/l)*	47.0	(32.8,64.6)	77.3	(48.1,102.0)	<0.001
Total Cholesterol (mmol/l)	5.8	1.1	5.3	1.2	0.12
HDL-Cholesterol (mmol/l)	1.6	0.4	1.3	0.3	0.007
LDL-Cholesterol (mmol/l)	3.7	0.9	3.3	1.0	0.039
Triglycerides (mmol/l)*	1.0	(0.7,1.3)	1.5	(1.0,2.4)	<0.001
zMS	-0.40	0.49	0.34	0.44	<0.001
Taking statin (%)	4 (9.8%)		10 (34.5%)		0.011
Taking ACEI/ARB (%)	7 (17.1%)		7 (24.1%)		0.47
Taking Betablocker (%)	5 (12.2%)		7 (24.1%)		0.19
Taking CCB (%)	4 (9.8%)		6 (20.7%)		0.20
Family History of DM (%)	10 (25%)		7 (25.9%)		0.93
Current or ex-smoker (%)	12 (29.3%)		12 (41.4%)		0.29

Continuous variables are summarised as means ± standard deviations, with p-values from t-tests. Continuous variables asterisked (*) have skewed distributions, so are summarised are medians and interquartile ranges, with p-values from Wilcoxon rank sum tests. Binary variables are summarised as N and %, with p-values from chi-squared tests.

ACEI – Angiotensin Converting Enzyme Inhibitor; ARB – Angiotensin Receptor Blocker; CCB – Calcium Channel Blocker; DBP – Diastolic Blood Pressure; DM – Diabetes Mellitus; HDL – High Density Lipoprotein; HIE – Hepatic Insulin Extraction; IHL – Intrahepatic Lipid; LDL – Low Density Lipoprotein; OGIS – oral glucose insulin sensitivity; SBP – Systolic Blood Pressure; zMS – Standardised Composite Metabolic Risk.

**Figure 1:**
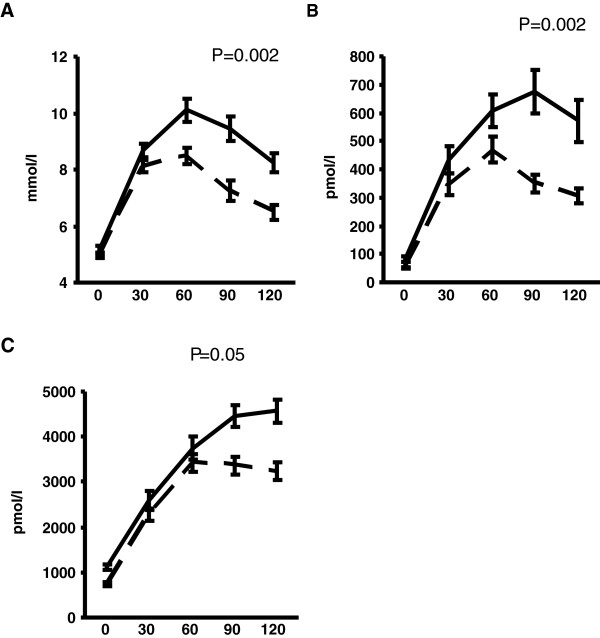
**(A-C): Glucose, insulin and C-Peptide levels during the oral glucose tolerance test.** Data are presented as mean ± SE. Dashed lines represent participants with IHL ≤ 5.5%. Solid lines represent participants with IHL > 5.5%, i.e. NAFLD. P values are derived from linear regression modelling with the exposure IHL treated as a continuous variable and the outcome being the area under the curve for the relevant metabolite, adjusted for age, gender, alcohol consumption and visceral fat area.

**Figure 2:**
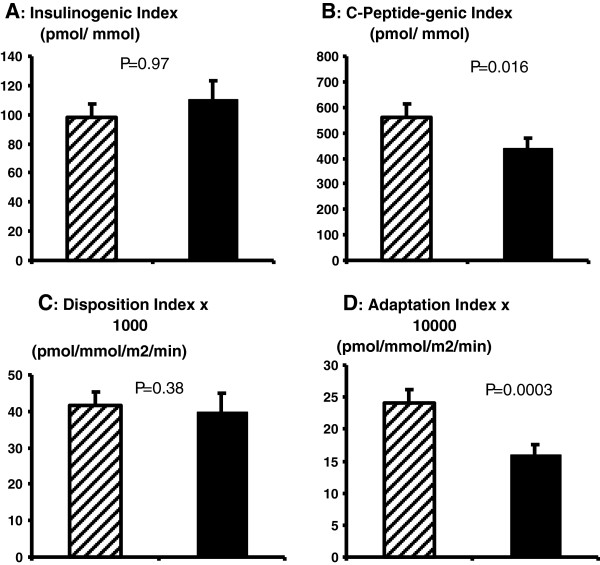
**(A-D): OGTT-derived indices of beta-cell function.** Data are presented as means ± SEM. Dashed bars represent participants with IHL ≤ 5.5%. Solid bars represent participants with IHL > 5.5%, i.e. NAFLD. P values are derived from linear regression modelling with the exposure IHL treated as a continuous variable, adjusted for age, sex, visceral fat area and alcohol consumption.

Increased IHL was associated with greater areas under the curve (AUCs) for glucose, insulin and C-Peptide during the OGTT, as shown in Figure 1 (A-C). There was a relatively small but nonetheless statistically significant inverse association between IHL and HIE (mean HIE 88.1 ± 0.5 v 85.6 ± 0.9% in those with normal IHL and elevated IHL, respectively: adjusted β = -0.2 [-0.3, -0.06], p < 0.006). Put another way, every 1% rise in IHL was associated with a 0.2% reduction in HIE. Although there was an absolute increase in insulin secretion with higher IHL, there were no significant associations between IHL and insulin-derived indices of beta-cell function (insulinogenic index and disposition index – IGI, DI) as shown in Figure 2 (A and C). However, there were inverse associations between IHL and C-Peptide derived measures of beta-cell function (C-Peptide-genic index and adaptation index – CGI and AI), as shown in Figure 2 (B and D), such that higher levels of liver fat accumulation were associated with less beta-cell C-Peptide secretion. After adjusting for insulin sensitivity measured by OGIS, the strength of evidence for this association was slightly diminished (beta = -11.5 [-23.7, 0.59], p = 0.062).

## Discussion

These data indicate for the first time that IHL accumulation is associated with impaired beta-cell function in healthy older individuals. The inverse associations between liver fat and some, but not all parameters of beta cell function are of particular interest, because they challenge the traditional notion that NAFLD is associated only with impaired insulin action and clearance [1,19]. We found that insulin and C-Peptide secretion after oral glucose loading were higher in participants with increased IHL. However, when their secretion was quantified in the context of dynamic glucose excursions after oral glucose loading and after further consideration of peripheral insulin sensitivity, an altogether different physiological pattern emerged: Increased IHL was associated with reduced rather than increased beta-cell function according to C-Peptide based model parameters (CGI and AI). The absence of any association with the parameters derived from insulin rather than C-Peptide may be due to the decreased HIE observed with increased IHL. This would not influence C-Peptide levels which are excreted by the kidneys. Our findings suggest that beta cell dysfunction may be an additional contributor to dysglycaemia in NAFLD but that reduced HIE can mask this phenomenon.

The association between liver fat and reduced HIE is consistent with previous observations in younger individuals [5]. However when Mehta et al examined differences in HIE in men with NAFLD compared to those with low normal liver fat content (IHL < 1%), there was a trend towards a difference (83 v 76%, respectively) but it was not statistically significant. It may be that by treating IHL as a continuous rather than a categorical variable in our analyses, we had greater statistical power to detect a difference in HIE, even though it was a smaller one (88 v 86%).

The same pathophysiological processes that give rise to accumulation of ectopic fat within the liver are likely to account for impaired beta-cell function also [7]. It is likely that our measures of liver fat reflect ectopic fat deposition in other non-adipose tissues, including the pancreas. Clearly, direct measures of pancreatic fat content would add strength to our study but were not available. However, studies that have examined the relationship between pancreatic fat content and beta-cell dysfunction have not been entirely conclusive to date. A study of 64 overweight adults with a family history of type 2 diabetes showed that pancreatic fat was associated with whole body insulin sensitivity and with glucose tolerance status, but not with beta-cell function per se [8]. Participants in that study were carefully assessed with gold-standard measures including hyperinsulinaemic clamp and the arginine stimulation test, but the relatively specific phenotype may have narrowed the metabolic variability of the cohort and limited statistical power again. Further studies to determine the influence of pancreatic fat content on insulin secretion would be helpful. Additionally, the association between excess fat accumulation and insulin resistance is known to be mediated by inflammatory processes [20], but we did not measure indices of inflammation here. This is a potentially important area for future studies to consider.

It was notable that the association between IHL and CGI no longer achieved an arbitrarily defined threshold for statistical significance after adjusting for OGIS, when p = 0.062. However, we do not believe that this negates our finding that IHL influences beta cell function. AI and CGI were both convincingly inversely associated with IHL and while it would have been even more convincing if the association with CGI remained significant after adjusting for insulin sensitivity, the fact that it didn’t does not nullify our findings. A key consideration here is the inverse colinearity between IHL and OGIS, which is well established. It may be that the observed association between IHL and reduced beta cell function is mediated entirely by insulin resistance. However, one would anticipate that the directions of association with insulin resistance would be opposite for beta cell function and liver fat content. Moreover, insulin resistance is not thought to influence beta cell function per se, it just leads to more insulin being produced. So, if insulin resistance was directly (as opposed to inversely) associated with beta cell function, then it might be reasonable to insist that any assessment of the strength of the association between liver fat (which is known to cause insulin resistance) and beta cell function would need to adjust for insulin resistance. We don’t believe such an approach is necessary here, though further studies would clearly be helpful.

Our study has a number of important strengths. Very detailed anthropometric and metabolic characterisation was conducted in each participant. MR spectroscopy is the most robust non-invasive method for quantifying IHL. Rather than comparing categories of steatosis, body fatness or glycaemic status, as many studies do, all our measures are continuous and represent the distributions in a relatively healthy cohort of older white participants. The study also has some limitations, not least that it was a post-hoc analysis of data from a cohort subgroup who were willing to participate in an exercise trial. All participants in the trial were white. Thus, our results may not be generalisable to all older people. Also, only 70% of people who enrolled in the trial had MR imaging at baseline, while others were unwilling or were too large for the scanner, which may have introduced bias. Given the relatively small size of our study population of older white exercise programme participants, confirmatory studies in other populations seem warranted.

There was a substantial level of “sub-clinical” metabolic disturbance in this cohort, particularly in relation to the number of individuals with abnormal glucose metabolism during the OGTT. It is important to note that these abnormalities were only detected through participation in the study and were not diagnosed prior to it (and so were not “prevalent” as such). All of these individuals volunteered to participate in a twelve-week exercise intervention. Diabetes was one of several exclusion criteria. Nonetheless, 43% had some form of abnormal glucose metabolism. None had symptoms of hyperglycaemia, nor were they on treatment for it at the time of testing. Within a cohort of this age a certain level of undiagnosed metabolic disease, be it diabetes or liver steatosis, is to be expected, so to be able to quantify this so precisely contributes to the novelty of our findings. We have kept variation in important but mechanistically less relevant confounders such as age and ethnicity to a minimum, while incorporating broader distributions of more relevant confounders such as adiposity, insulin sensitivity and liver fat content.

## Conclusion

In our experience, there is a widely held perception that liver steatosis is associated with increased production of insulin from the beta-cell in order to compensate for whole-body insulin resistance. However, the elevation in insulin levels seen in those with NAFLD is misleading and is explained by their reduced hepatic insulin extraction. We have shown for the first time in apparently healthy older adults that liver steatosis is associated with a relative defect in beta cell function. We have been careful to take account of confounding factors such as age [21], sex [22] and alcohol consumption. Further studies quantifying pancreatic fat content directly and its influence on beta cell function would be valuable in elucidating further the mechanistic basis for the development of type 2 diabetes.

## Abbreviations

AI: Adaptation index; CGI: C-peptide-genic index; DI: Disposition index; HIE: Hepatic insulin extraction; HPAT: Hertfordshire physical activity trial; IGI: Insulinogenic index; IHL: Intrahepatic lipid; NAFLD: Non-alcoholic fatty liver disease; OGIS: Oral glucose insulin sensitivity; VAT: Visceral adipose tissue.

## Competing interests

The authors declare that they have no competing interests.

## Authors’ contributions

F.M.F. contributed to study design, data acquisition, analysis and interpretation and writing the manuscript. S.J.S. contributed to study design, data analysis and interpretation and writing the manuscript. M.H. contributed to data interpretation and writing the manuscript. A.S. contributed to study design, data acquisition and analysis and writing the manuscript. E.D.L.R. contributed to study design, data acquisition and analysis and writing the manuscript. A.A.S. contributed to study design, data acquisition and writing the manuscript. C.C. contributed to study funding, design, data acquisition and writing the manuscript. S.J.G. contributed to study funding, design, data acquisition, analysis and interpretation and writing the manuscript. N.J.W. contributed to study funding, design, data acquisition, analysis and interpretation and writing the manuscript. All authors read and approved the final manuscript.
